# Organizational dynamics and individual motivations in VR integration in higher education

**DOI:** 10.1186/s12909-026-09911-3

**Published:** 2026-07-09

**Authors:** Frode Johansen, Maria Løvsletten, Helge Toft, Odd Rune Stalheim

**Affiliations:** 1 Section for Nursing, Faculty of Social and Health Sciences, University of Inland Norway, Terningen Arena, Hamarveien 112, Elverum, 2406 Norway; 2 Section for Mental Health and Rehabilitation, Faculty of Social and Health Sciences, University of Inland Norway, Terningen Arena, Hamarveien 112, Elverum, 2406 Norway; 3 Department of Educational Studies in Teacher Education, Faculty of Education, University of Inland Norway, Midtbyen Park, Skoleveien 4, Hamar, 2318 Norway; 4 Innlandet Hospital Trust, Furnesvegen 26, Brumunddal, 2380 Norway

**Keywords:** Virtual reality (VR), Higher education, Diffusion of innovations, Motivated reasoning, Technology adoption, Organizational dynamics, Qualitative research, Thematic analysis

## Abstract

**Background:**

Virtual Reality (VR) is increasingly adopted in higher education, offering immersive learning experiences that bridge theoretical knowledge and practical application. However, its integration is influenced by organizational dynamics and individual motivations, requiring a deeper understanding of these factors to optimize its potential.

**Methods:**

This qualitative study employed thematic analysis of semi-structured interviews with four educators from health and social care programmes at three universities and one educator from a police education programme. Participants shared their experiences integrating VR into curricula, focusing on leadership, collaboration, training, and resource allocation. Data were analysed using Braun and Clarke’s reflexive thematic analysis, supported by F4 Analysis software, to identify key themes related to VR adoption and implementation.

**Results:**

Six themes emerged, highlighting both enablers and barriers to VR adoption. Leadership support and motivated early adopters were critical for initial implementation, but challenges included high costs, inadequate training, and limited alignment with curricula. Collaboration, both internally and with external partners, facilitated tailored VR content but required sustained effort and resources. Participants also noted that VR provided opportunities to enhance student preparedness for real-world scenarios, although its perceived complexity occasionally hindered broader acceptance. Despite variability in institutional readiness, a common need for ongoing professional development and strategic planning was evident.

**Conclusions:**

The findings underscore that successful VR integration in education relies on aligning technological innovations with pedagogical design and institutional strategies. Leadership support, robust collaboration, and sustained training are pivotal for overcoming barriers. These preliminary, hypothesis-generating insights point to factors that institutions may wish to consider when adopting VR, while underscoring the need to balance enthusiasm with critical evaluation.

**Supplementary Information:**

The online version contains supplementary material available at https://doi.org/10.1186/s12909-026-09911-3.

## Introduction and background

The current landscape of VR in education is characterized by rapid innovation and increasing integration into curricula. Institutions across the globe are exploring the potential of VR to enhance student engagement, improve learning outcomes, and provide personalized learning experiences [1, 2]. A growing trend in professional settings is the use of VR technology to create immersive simulations that aim to replicate real-world challenges. These simulations enable students to apply theoretical knowledge in practical settings, bridging the gap between theory and practice [3, 4]. Another emerging trend is developing VR content specifically designed to target and achieve defined educational outcomes. Unlike early VR applications, which were often generic and focused on novelty, the current generation of VR educational tools is designed with a pedagogical intent [5]. This pedagogical approach includes efforts to create VR modules tailored to the needs of different disciplines and learning levels [6–8], intending to make the technology not just an add-on, but an integral part of the educational process [9].

The adoption of VR in education is influenced not only by technological and financial factors but also by organizational dynamics and individual motivations. Teacher readiness and pedagogical competence have been identified as critical factors in determining the successful adoption of VR in educational settings, underscoring the importance of adequate planning and professional development initiatives [10]. Institutions that successfully integrate VR into their curricula often do so because of a combination of top-down support from leadership and bottom-up enthusiasm from educators [11]. Organizational culture plays a crucial role in this process; institutions that foster a culture of innovation and provide support for experimenting with new technologies are more likely to see successful VR integration [12]. Providing professional development opportunities and resources for teachers to become familiar with VR and AR technologies has been highlighted as a key enabler for promoting their classroom use [13]. Individual motivations are also critical. Early adopters of VR often drive its implementation within their institutions. Supporting these early adopters by allowing them time and resources to experiment with VR has been shown to facilitate successful implementation [14]. Early adopters may have a personal interest or expertise in technology and often act as advocates who help overcome resistance from more sceptical colleagues [15]. Understanding the dynamics between individual motivations and institutional support is essential for institutions seeking to implement VR effectively, as both structural support and individual initiative are necessary for successful adoption [16].

Against this background, the aim of this study was to explore how organizational dynamics and individual motivations shape the early adoption of VR in higher education. Specifically, the study was guided by the research question: How do organizational dynamics and individual motivations shape the early adoption of VR technology in higher education? Addressing this question can help institutions make more informed, strategic decisions when integrating new digital tools such as VR.

## Theoretical framework

In understanding the early adoption of VR in higher education, this study draws on two key theoretical perspectives: Diffusion of Innovations (DOI) theory and Motivated Reasoning (MR). These frameworks provide a suitable foundation for analysing the complex interplay between organizational dynamics and individual motivations influencing technology adoption in educational settings.

### Diffusion of innovations theory

DOI, developed by Everett Rogers [17], is a well-established framework for examining how new ideas and technologies spread within organizations and societies. According to Rogers, adopting an innovation follows a predictable pattern, with individuals categorized into groups such as innovators, early adopters, early majority, late majority, and laggards, depending on how quickly they embrace the new technology. In the context of VR adoption in higher education, DOI theory helps to elucidate the varying rates at which different institutions and individuals adopt this technology. Factors such as VR’s perceived relative advantage, compatibility with existing educational practices, complexity, and ability to observe and trial the technology play crucial roles in determining the speed and extent of adoption [18]. For instance, institutions with a strong culture of innovation and a clear alignment between VR and educational goals are likely to move quickly through the adoption process. Conversely, institutions that perceive VR as complex or misaligned with their pedagogical approaches may lag behind [19]. Moreover, DOI theory highlights the importance of social systems in the diffusion process. The adoption of VR is not only an individual decision but is also influenced by the broader organizational context, including leadership support, resource availability, and peer influence. For example, early adopters within an institution can act as change agents, promoting the technology to others and supporting efforts to overcome resistance. Understanding these dynamics is essential for developing strategies to facilitate the adoption and integration of VR across different educational contexts [18].

### Motivated reasoning

Motivated Reasoning (MR), as described by Kunda [20], offers a psychological perspective on how individuals process information and make decisions regarding new technologies. According to MR, individuals are not always purely rational in their decision-making processes; instead, their choices are influenced by their desires, goals, and pre-existing beliefs. Such cognitive processes can lead individuals to selectively gather and interpret information to support their preferred conclusions, often leading to decisions that align with their existing attitudes and interests. In the context of VR adoption in higher education, MR provides insights into how individual motivations and cognitive processes shape decision-making. For instance, educators who are enthusiastic about technology and innovation may be more likely to advocate for VR, interpreting its benefits more favourably while downplaying potential challenges. Conversely, those who are sceptical of technology or have had negative experiences with previous innovations may focus on the drawbacks of VR, reinforcing their resistance to its adoption. Such a perspective is particularly relevant for understanding the role of early adopters in the diffusion of VR. Early adopters are often driven by a strong belief in the potential of new technologies and are willing to take risks to implement them. Their motivation may lead them to actively seek out information confirming VR’s value, thereby influencing the broader adoption process within their institutions. Although MR was originally formulated as a general account of how goals and prior beliefs shape information processing, it has since been applied specifically to attitudes toward emergent technologies, where framing and motivation influence how people weigh potential benefits and risks [21]. This makes MR well suited to examining how educators’ motivations shape their appraisal of VR, complementing the more system-level lens of Diffusion of Innovations theory.

## Methods

### Research design

This study employed a qualitative research design, utilizing semi-structured individual interviews to explore the use of VR in higher education. Qualitative research was chosen to provide rich, detailed insights into participants’ experiences and perceptions [22]. Semi-structured interviews allowed for flexibility in questioning, enabling the researcher to probe deeper into specific areas of interest while maintaining consistency across interviews [23]. The topics covered in the interviews included: the introduction of VR in the institution, the decision-making process, the selection of VR technology and content, collaboration in the development of VR solutions, the implementation of VR in existing curricula, the training and support services provided, experiences with VR in education, the evaluation of VR’s effectiveness, and future plans for VR development and expansion. The study adopted a theoretically informed (abductive) reflexive thematic analysis [24, 25]. Coding remained close to the data and was not constrained by a pre-defined coding frame; at the same time, Diffusion of Innovations and Motivated Reasoning were used as sensitizing concepts that informed the analysis iteratively, shaping the interview guide, prompting attention to leadership, social systems and individual motivation during coding, and guiding the interpretation of patterns, rather than being applied only retrospectively in the discussion. Moving abductively between the data and these frameworks allowed both data-driven themes and theoretically meaningful interpretations to be developed.

The study involved five participants, four women and one man, aged approximately 35 to 50 years, all of whom had experience with VR technology in education through head-mounted displays (HMDs) but had used different software and therefore had varied experiences with content. One participant represented a police education program, while the other four came from three different universities, each offering health and social care education programmes. Health, social care and police education were chosen because, in the Norwegian context, these fields have the most established and documented experience with HMD-based VR and a strong simulation-based pedagogical tradition; this focus also bounds the transferability of the findings (see Limitations). All participants were involved in teaching and integrated VR into their educational practices to varying degrees, providing insights based on their direct experience with the technology in a pedagogical context. The interviews were conducted in February 2024.

Participants were eligible for inclusion if they (i) were educators or resource persons in higher education; (ii) had hands-on experience integrating head-mounted display (HMD) VR into teaching; and (iii) had been involved in decisions about, or the implementation of, VR in their programme. Individuals without direct teaching-related VR experience, and those whose use was limited to one-off demonstrations, were excluded.

As is appropriate for qualitative designs, no a priori statistical sample size calculation was performed; instead, the number of participants was guided by the principle of information power [26]: because the aim was narrow, the participants held highly specific and relevant experience, and the analysis drew on established theory and case-level depth, a small but information-rich sample was considered sufficient to address the research question.

### Recruitment

Recruitment involved contacting universities and educational institutions to identify individuals using or planning to use VR in their teaching. We used purposive, criterion-based sampling to identify information-rich cases. Candidate institutions were identified through publicly available information (institutional and project web pages and, in some cases, media reports of VR initiatives); media visibility was used only as one indicator to locate institutions with relevant experience, not as the selection criterion in itself. The process began by contacting department heads and programme coordinators in health, social care and police education programmes. These leaders identified candidates with direct experience of VR technology (specifically head-mounted displays) and asked whether they would be willing to be contacted about the study; where a candidate agreed, the leader shared the candidate’s email address with the first author, who then approached them with comprehensive information about the study, including its aims, procedures, and ethical considerations. Participants were also informed that the interviewer was a PhD candidate exploring VR adoption in higher education. The interviewer had no prior personal or professional relationship with any of the participants. All invited participants agreed to take part, and no one withdrew from the study.

### Data collection

Data were collected through individual interviews conducted via Microsoft Teams [27]. All interviews were conducted by the first author (FJ), a male registered nurse who had qualified 25 years earlier. At the time of the study, FJ held a full-time PhD-candidate position with no teaching duties and had received training in qualitative methods and interview techniques during both his master’s and doctoral programmes. Only the interviewer and the participant were present during each interview. No repeat interviews were conducted, no field notes were taken, and transcripts were not returned to participants for comment. The interviews lasted between 40 min and one hour. Audio recordings were made using Nettskjema, a data-collection service operated by the University of Oslo on secure research infrastructure, ensuring that no data were stored on the recording device. The recordings were automatically transcribed in Nettskjema, and the transcripts were then manually checked for accuracy. The semi-structured interview guide was developed specifically for this study. Questions were derived from the research question and from prior literature on technology adoption and VR in education, and were organized around leadership and decision-making, technology and content selection, collaboration, curriculum integration, training and support, and future plans. The guide was reviewed for clarity and relevance by all members of the research team and refined after a pilot interview; minor wording changes were made, and no major revisions were required. Because the guide is an interview aid rather than a psychometric instrument, formal scale validation was not applicable; trustworthiness was instead supported through team review, piloting, and consistent use of the guide across interviews.

### Data analysis

The data analysis followed Braun and Clarke’s reflexive thematic analysis [24], facilitated by the F4 Analysis software [28]. This methodology enabled the identification of key themes surrounding the use and integration of VR in higher education. Throughout the process, the interview guide was referenced to ensure consistency and alignment with the research objectives. The six phases began with familiarization with the data, where the researcher immersed themselves in the transcripts, reading, and re-reading to get a holistic understanding, thereafter, followed by generating initial codes, where meaningful features of the data were systematically highlighted. After initial coding, the next step involved searching for themes, where related codes were grouped into potential themes. While reviewing themes, the themes were refined by checking them against the coded data and the entire dataset to ensure coherence. This process was iterative, involving back-and-forth analysis between the data as a whole and the specific details within the themes. In the next phase, the researchers focused on defining and naming themes, ensuring that each theme was distinct and contributed to the overall understanding of the data. Finally, the themes were presented in a cohesive narrative as part of the production of the final report, highlighting insights into the adoption and use of VR in higher education.

The first author conducted the initial coding and developed the candidate themes. The coding tree and emerging themes were then reviewed and challenged by the co-authors in several analytic meetings; interpretive differences were discussed until consensus was reached, and themes were refined against the full dataset. Consistent with a reflexive thematic analysis approach, themes were understood as analytic constructions developed through interpretation rather than as outputs requiring inter-coder reliability statistics. The team’s mixed disciplinary backgrounds (nursing, mental health, and education) supported reflexivity throughout. The first author approached this work with a balanced view of digital technology in education, positive toward technological development while attentive to the need for critical evaluation, and this stance was acknowledged as a potential influence on analytic interpretation and addressed through the team-based discussion of coding described above. Consistent with this approach, member checking was not undertaken; participants did not provide feedback on the findings. This study is reported in accordance with the Consolidated Criteria for Reporting Qualitative Research (COREQ). The completed COREQ checklist is provided as a supplementary file.

Through this iterative process, six main themes emerged: Leadership and Strategic Considerations in Early VR Adoption; Exploring How VR Both Addresses and Challenges Educational Needs; Collaborative Approaches to Navigating Barriers and Opportunities in VR Adoption; Navigating Organizational and Technical Complexities in VR Integration; Considering Future Possibilities and Limitations for VR in Higher Education; Reflections on Key Insights for VR Adoption in Education. These themes were identified as they represent the most substantial and recurring patterns in the material, offering a comprehensive view of how VR technology is perceived, integrated, and adapted within educational settings. Each theme captures distinct but interconnected aspects of the participants’ experiences, reflecting both individual and organizational perspectives. The thematic analysis process allowed for a flexible yet systematic approach, ensuring that the themes provided a descriptive and meaningful representation of the core insights drawn from the data.

### Ethical considerations

All participants were provided with detailed information about the study both verbally and in writing, emphasizing that their participation was entirely voluntary. They were informed of their right to withdraw from the study at any time without any consequences and could request the deletion of their contributed data. Signed consent forms were obtained prior to conducting the interviews. The study received approval from the Committee for Research Ethics (KOFE) at Inland Norway University of Applied Sciences, ensuring compliance with all relevant ethical standards and guidelines (Approval No. 18-2023; Archive: 2023/05337). The research was conducted in accordance with the ethical principles of the Declaration of Helsinki [29]. The confidentiality and anonymity of participants were maintained throughout the research process. Given the small sample size, and particularly the fact that only one participant represented the police education program, additional measures were taken to protect participant anonymity. Specifically, the participant numbers used in the results section were not linked to their specific educational affiliation to ensure that no individual could be easily identified based on their responses or professional background.

## Results

This section presents the findings, structured according to the six main themes identified during the thematic analysis. Each theme captures key insights related to the adoption and use of VR in higher education, based on participant experiences and reflections. These themes reflect both the opportunities and challenges of integrating VR technology into educational practices, ranging from strategic leadership considerations to the practicalities of implementing VR across different disciplines. The following subsections will elaborate on these themes, supported by participant quotes to provide a nuanced understanding of their experiences.

Identified themes, sub-themes, and codes are summarized in Table 1, where the numbers in parentheses represent the frequency of codes identified for each category. These counts are descriptive and should not be interpreted as participant counts or as measures of theme importance. For instance, under the sub-theme ‘Start-up and initial considerations,’ the code ‘Initial steps’ (9) includes participant statements such as: “We needed to ensure that everyone understood the basic functionality of the VR equipment before moving forward”.

**Table 1:** Identified themes, sub-themes, and codes

Theme	Sub-theme	Codes
Leadership and Strategic Considerations in Early VR Adoption (51)	Start-up and Initial Considerations (26)	Initial Steps (9), Organization (9), Leadership (8)
	Pilot Projects and Feasibility Studies (14)	Relevance (8), Curiosity (6)
	Risk and Uncertainty Management (11)	Evaluations (11)
Exploring How VR Both Addresses and Challenges Educational Needs (40)	Identified Educational Needs (13)	Reflections on Needs (13)
	Stakeholder Expectations and Concerns (4)	Potential Contributions (4)
	Alignment with Learning Outcomes (23)	Structuring (15), Pedagogy (8)
Collaborative Approaches to Navigating Barriers and Opportunities in VR Adoption (34)	External Partnerships and Vendor Relationships (12)	Nature of Collaboration (12)
	Intra-organizational Collaboration (22)	Colleagues (18), Leadership (4)
Navigating Organizational and Technical Complexities in VR Integration (102)	Curriculum Integration and Challenges (26)	Opportunities for VR in Education (9), Strategies and Plans (17)
	Faculty Training and Support (27)	Technological Challenges (4), Support Functions (2), Anchoring and Structure (21)
	Student Engagement and Interaction (28)	Experiences and Reflections (22), Integrated into Education (6)
	Assessment and Review (21)	Informal Feedback (13), Metrics and Scores (8)
Considering Future Possibilities and Limitations for VR in Higher Education (29)	Collaboration for Resource Sharing (8)	Outreach Activities (5), Sharing (3)
	Technological Innovation and Adaptation (17)	Ambitions (8), Internal Plans (9)
	Expansion to New Educational Domains (4)	
Reflections on Key Insights for VR Adoption in Education (29)	Required Adjustments (3)	
	Key Takeaways for Future Efforts (10)	Organization (4), Pragmatism (6)
	Success Factors (16)	User Orientation (5), Anchoring (4), Collaboration (3), Forward-Thinking (4)

### Leadership and strategic considerations in early VR adoption

There was broad agreement among the participants that the early adoption of VR in education was driven by exploratory efforts and proactive individuals. In addition, leadership played an important role, although most participants acknowledged the lack of formal assessments and structured evaluations as a common challenge.

The introduction of VR technology was marked by a combination of exploratory efforts and foundational assessments aimed at understanding its potential within the educational context. Informant 1 described this phase as a period of investigation: “It started as a pre-project aimed at determining whether VR could enhance learning outcomes for students, particularly given the limited time available in our clinical simulation labs.” Informant 1 further emphasized that the initial explorations were necessary to establish whether VR could be realistically integrated into the existing educational structure: “We had to consider if this technology would be sustainable in the long run, not just a passing trend.” On an individual level, Informant 5 highlighted personal initiative as a key driver: “I was the one who introduced the idea after researching developments globally and identifying VR as an emerging tool in nursing education.” The role of motivated individuals in driving early adoption within the organization is emphasized through this proactive approach. Despite these efforts, there was some variability in how thorough the early assessments were perceived. While some informants felt that the groundwork was sufficient, others noted the absence of a formal, systematic analysis. Informant 4 remarked: “We didn’t conduct a systematic needs analysis; it was more about informal conversations with staff and exploring existing literature.” Informant 2 echoed this sentiment: “We were eager to innovate, but in hindsight, we might have benefited from a more structured approach to evaluating the feasibility of VR in our context.”

### Exploring how VR both addresses and challenges educational needs

Participants generally agreed that VR can potentially address educational gaps, particularly in simulation-based training. Participants also expressed mixed views regarding its alignment with existing curricula, and several informants raised concerns about its effectiveness for more advanced levels of education.

Expectations and perceived needs were a recurring theme in shaping the adoption and implementation of VR technology. Informants consistently emphasized the potential of VR to address specific gaps in the current educational offerings, particularly in areas requiring simulation and hands-on practice. Informant 2 noted: “We had high expectations that VR would allow students to practice scenarios that are difficult to replicate in real-life settings, such as emergency procedures and complex patient interactions.” Several informants also mentioned that these expectations were rooted in the desire to better prepare students for real-world challenges. However, these expectations were not uniform. Informant 4 pointed out that while the technology was promising, there was a degree of uncertainty regarding its alignment with existing learning objectives: “We weren’t entirely sure how VR would fit into the established curriculum and whether it would truly meet our learning objectives without significant adjustments.” Informant 1 also expressed caution: “While we were excited about the potential of VR, we had to be realistic about its limitations and ensure that it would complement, rather than complicate, the educational framework.” Moreover, the needs assessment revealed that while VR had clear potential in some areas, such as first-year training, its applicability to more advanced levels of study was questioned. Informant 4 observed: “The VR scenarios were well-suited for first-year students, but more advanced students found the tasks too basic, which raised concerns about its broader utility across different levels of education.”

### Collaborative approaches to navigating barriers and opportunities in VR adoption

There was consensus that collaboration—both internally and with external partners—was critical to the successful implementation of VR. Although participants recognized the importance of these partnerships in adapting VR content, several noted that maintaining these collaborations over time was a significant challenge.

Collaboration, both within the institution and with external partners, emerged as a key factor in successfully implementing VR. Informant 3 highlighted the importance of this collaborative approach: “Our project group was composed of individuals with both technical and pedagogical expertise, which allowed us to address the various challenges that arose during the implementation process.” Informant 1 stated: “Having a committed team from different departments ensured that we could tackle problems from multiple angles and keep the project moving forward.” Externally, collaborating with the VR software provider was essential in adapting the technology to meet the institution’s specific needs. Informant 5 mentioned: “We had ongoing discussions with the software provider to ensure that the VR content was culturally and contextually relevant for our students, which included adapting certain procedures to reflect Norwegian healthcare standards.” This collaboration was instrumental in customizing the technology and ensuring its relevance to the local context. Nonetheless, challenges were noted in maintaining these collaborative efforts over time. Informant 4 observed: “While the initial collaboration was strong, sustaining it required continuous effort and resources, which became challenging as the project expanded.” Informant 2 also reflected on the challenges of maintaining long-term partnerships: “It’s one thing to start a collaboration but ensuring that both sides remain engaged and committed as the project evolves is a different challenge altogether.”

### Navigating organizational and technical complexities in VR integration

Most participants agreed that VR integration faced organizational and technical challenges, particularly due to inconsistent use, reliance on key individuals, limited evaluation practices, and the need for broader faculty support.

The implementation of VR technology faced several challenges, particularly regarding its integration into the existing curriculum. Informant 3 remarked: “The headsets have spent more time in a cupboard than in use; it’s been difficult to make it a consistent part of the curriculum.” As informant 2 highlighted, this reflects a broader issue in technology adoption, where the initial excitement can wane if the technology is not seamlessly integrated into regular educational practices: “We needed to think more about how VR would fit into the broader educational strategy, not just as an add-on, but as an integral part of the learning experience.” The reliance on a few key individuals to promote the technology also posed challenges. Informant 4 explained: “The project heavily relied on a few key individuals who were enthusiastic about VR. Without their continuous involvement, it has been hard to sustain momentum and ensure that the technology is used effectively across different courses.” Informant 1 reiterated the importance of having dedicated personnel: “In the early stages, having proponents for VR was essential. These individuals drove the project forward and ensured that others saw its potential.” In addition, the integration process highlighted the need for more extensive training and support for faculty members. While some training was provided, it was mainly limited to introductory sessions. Informant 4 emphasized: “We provided some group training and developed a few introductory videos, but there is a clear need for more systematic support to empower staff to use the technology independently.” Informant 2 noted that this collective support was a factor for integrating VR: “Without ongoing training and resources, it’s difficult for faculty to feel confident in using VR as a teaching tool.”

Assessment and review also emerged as part of these organizational and technical complexities. Participants described evaluation practices as mainly informal and exploratory rather than as systematic assessments of learning outcomes. Informal feedback included comments from students and staff after VR sessions, particularly concerning usability, engagement, and perceived relevance to the curriculum. Some participants also referred to metrics and scores generated within the VR applications, although these were used primarily as practical feedback for further development and reflection rather than as validated measures of educational effectiveness.

### Considering future possibilities and limitations for VR in higher education

There was general optimism about the future potential of VR, with most participants agreeing that further development of more advanced scenarios and stronger institutional collaboration is needed to fully realize its benefits.

There is a strong consensus among informants that VR has significant potential to enhance education, particularly in areas that are difficult to address through traditional methods. Informant 1 expressed optimism about the future of VR: “We see great potential in using VR for training scenarios that are difficult to replicate in real-life practice, such as specific mental health situations and emergency procedures.” Such a forward-looking perspective is shared by other informants, who see VR as a valuable tool for addressing specific educational challenges. However, there is also a recognition of the need for further development and refinement of the technology. Informant 4 noted: “While VR has great potential, it’s clear that we need to develop more advanced and interactive scenarios to fully realize its benefits, particularly for more experienced students.” Informant 2 echoed this, emphasizing the importance of tailoring VR content to different learning levels: “As we move forward, it will be crucial to ensure that VR can cater to both novice and advanced students, providing them with appropriate challenges.” Additionally, there is a growing interest in exploring collaborative opportunities with other institutions to share resources and expertise. Informant 5 proposed: “It would be immensely beneficial if we could create a common repository where educational institutions can share their VR resources and build on each other’s work.” Informant 1 also supported this idea, stating: “Collaborating with other institutions could accelerate the development of high-quality VR content and ensure that we’re not duplicating efforts.”

### Reflections on key insights for VR adoption in education

Participants widely agreed that aligning VR with specific educational goals and providing ongoing training and support for faculty are essential. There was also consensus that sustained collaboration and strategic planning are critical for VR’s long-term, effective use in education.

Implementing VR technology in education has provided participants with valuable insights into the challenges and opportunities of adopting new technologies. One of the key experiences shared by participants is the importance of aligning technological innovations with the specific needs and goals of the educational program. Informant 4 reflected on this: “One of the biggest takeaways for us is that while VR has a lot of potential, it needs to be closely aligned with our learning objectives to be truly effective.” Another notable lesson is the need for sustained support and training for faculty members. Informant 3 emphasized: “We’ve learned that it’s not enough to simply introduce new technology; we need to provide ongoing support and training to ensure that it’s used effectively and becomes an integral part of our educational practices.” Informant 1 added that continuous learning is crucial: “The technology is evolving rapidly, and so must our approach to training and support. We need to stay ahead of the curve.” All the informants emphasized the importance of collaboration, within the institution and with external partners. Informant 5 noted: “Collaborating with our VR provider was crucial in ensuring that the technology met our needs, and I believe that continued collaboration will be key to sustaining and expanding our use of VR in the future.” Such a statement suggests that building strong partnerships and fostering a collaborative culture are essential for successfully adopting new technologies in education.

## Discussion

The findings of this study highlight the complex interplay between organizational dynamics and individual motivations in the early adoption of VR technology in higher education. Grounded in Diffusion of Innovations and Motivated Reasoning theories, this article offers a nuanced understanding of how educational institutions and their members approach new technological tools like VR.

### Understanding organizational dynamics

The study underscores that organizational readiness plays an important role in adopting VR technology. Institutions with a clear strategic vision for integrating new technologies tend to be more successful in adopting VR. This finding is consistent with previous research, such as that by Arundel, Bloch, and Ferguson [30], emphasizing the importance of aligning technological innovations with institutional goals to ensure successful adoption. The Diffusion of Innovations theory supports this by highlighting the role of perceived relative advantage and compatibility in influencing adoption decisions. This study highlights that strong leadership, and a committed team of educators are crucial for advancing VR within the institution. Participants emphasized the importance of leadership support in fostering innovation and driving the implementation of new technologies. However, the findings also reveal that a lack of systematic needs analysis may create a disconnect between enthusiasm for innovation and practical implementation, potentially jeopardizing long-term sustainability [31]. These findings align with Rogers’ [17] argument that without a thorough assessment of the innovation’s attributes, such as compatibility and complexity, technology adoption can lead to inefficiencies and underutilization, particularly in the later stages of the diffusion process. The results indicate that institutions must take a more pragmatic approach, ensuring that new technology closely aligns with their educational goals and existing infrastructure. Furthermore, ongoing institutional support, both in terms of resources and training, is vital for maintaining momentum in VR integration. The importance of continuous support shows that technology adoption is more sustainable when there is a structured plan for ongoing training and resource allocation, as also found by Hughes et al. [32].

### The role of individual motivations

On an individual level, the study reveals that personal initiative and motivation are significant drivers in the early stages of VR adoption. This aligns with research by Stranger-Johannessen and Fjørtoft [33], emphasizing the role of early adopters in driving technological change within educational settings, aligning with the principles of Diffusion of Innovations (DOI) theory. Participants who were proactive in researching and advocating for VR played a crucial role in bringing the technology into their institutions. However, Motivated Reasoning theory suggests that these individuals may also selectively process information that supports their enthusiasm for VR, potentially overlooking challenges or limitations [20]. The tension between innovation and practicality was evident in the varied expectations among participants. While some viewed VR as a groundbreaking tool that could revolutionize education, others were more cautious, recognizing the need for careful alignment with curriculum goals. This divergence highlights the importance of balanced decision-making that combines enthusiasm with critical evaluation; where such balance is achieved, innovation is more likely to be aligned with educational objectives. This interpretation is consistent with work on framing and motivated reasoning about emergent technologies [21].

### Collaboration and customization

The findings emphasize the importance of collaboration, both within the institution and with external partners, in successfully implementing VR. Internal collaboration across departments facilitated a more holistic approach to integrating VR, ensuring that both technical and pedagogical aspects were considered. Such an approach aligns with research showing that interdisciplinary collaboration enhances the effectiveness of technology integration in education [34]. This study found that collaborating closely with VR software providers enabled institutions to tailor the technology to their specific needs, a factor identified as crucial for its acceptance and effectiveness [35]. However, sustaining these collaborative efforts over time proved challenging. The study highlights the need for long-term partnerships and continuous engagement with stakeholders to ensure that VR remains relevant and effective. Amey and Eddy [36] found that the success of educational technology projects often depends on the strength and longevity of partnerships. Institutions must therefore consider how to build and maintain these relationships to support the ongoing development and refinement of VR applications. Research also highlights that robust partnerships are key to the continuous improvement and adaptation of educational technologies [37].

### Theoretical contribution

Beyond applying these frameworks, our findings also refine them. They extend Diffusion of Innovations by showing that, in the earliest phase of adoption, the ’change agent’ role identified by Rogers [17] may be concentrated in one or very few individuals, making the diffusion process fragile: when these individuals disengage, diffusion can stall even where relative advantage is recognized. The findings also suggest that motivated reasoning operates not only at the individual level described by Kunda [20] but also collectively, as enthusiasm and selective attention to VR’s benefits become shared within project groups. Together, these observations point to a need to theorize early technology adoption as a socially distributed process in which individual motivation and organizational structure are tightly coupled.

### Practical implications and future directions

The study suggests that institutions should adopt a strategic approach to VR integration, beginning with a thorough needs analysis and aligning the technology with clear educational objectives. Such an approach aligns with recent work on aligning technology initiatives with institutional priorities [38]. Institutions should also invest in ongoing professional development to equip educators with the skills and confidence to use VR effectively. The importance of professional development is underscored by research, which shows that continuous training is crucial for successfully adopting and integrating new technologies in education [39]. Looking forward, developing more advanced and interactive VR scenarios will be crucial to expanding its applicability across different levels of education. Di Natale et al. [40] support this perspective, advocating for the continuous evolution of educational technologies to meet the changing needs of learners. Additionally, fostering collaborative opportunities with other institutions could enhance the quality and breadth of VR content, ensuring that resources are shared and efforts are not duplicated. Cook et al. [41] highlight the importance of this collaborative approach, finding that inter-institutional collaboration can significantly enhance the effectiveness of educational technology initiatives.

Future research should further explore the role of individuals in the early stages of VR implementation, as the findings from this study suggest that the process often depends heavily on one or very few individuals to initiate and champion the integration of VR. This reliance could pose a potential vulnerability, as the success and sustainability of VR adoption may depend on these individuals’ ongoing engagement and influence. Investigating how institutions can more effectively distribute this responsibility across teams, foster broader buy-in, and ensure resilience in implementation efforts would provide valuable insights for advancing the integration of VR in higher education. Future research could also apply Activity Theory to analyse individual institutional cases, making visible the contextual strengths and contradictions that shape VR adoption within each activity system; such an approach has proved fruitful for studying technology integration in education [42].

### Limitations

While this study provides valuable insights into the early adoption of VR in higher education, several limitations should be acknowledged. The small sample size and the focus on institutions with prior experience in VR may limit the generalisability of the findings to broader educational contexts. In addition, our recruitment strategy targeted institutions that were already visible through project pages and media coverage, as experienced VR users. This may have introduced a selection bias toward more successful or enthusiastic cases and toward more positive accounts of VR adoption, and it further limits the transferability of the findings to institutions at earlier or less visible stages of adoption. Only one participant represented police education, which may have skewed the diversity of perspectives captured. Additionally, the reliance on semi-structured interviews introduces potential biases, as participants’ self-reported experiences may have been influenced by individual enthusiasm or dissatisfaction, emphasizing either the positive or negative aspects of VR integration. Finally, the study did not include longitudinal data, which limits the ability to assess the long-term sustainability and impact of VR adoption on teaching practices and learning outcomes.

## Conclusion

This study set out to answer the research question: How do organizational dynamics and individual motivations shape the early adoption of VR technology in higher education? The findings demonstrate that the integration of VR is a multifaceted process significantly influenced by both institutional structures and individual initiatives. Organizational dynamics, such as leadership support, strategic alignment with institutional goals, and the availability of technical and pedagogical resources, were found to be critical enablers of early adoption. At the same time, individual motivations, particularly those of early adopters who act as advocates of the technology, were instrumental in initiating and sustaining its implementation. The study highlights that successful adoption requires the alignment of these factors. While motivated individuals can drive early adoption through enthusiasm and advocacy, their efforts must be reinforced by strong institutional leadership, clear strategic planning, and robust collaborative frameworks. Without this alignment, the implementation of VR risks becoming inconsistent and unsustainable. Moreover, challenges such as high costs, insufficient training, a lack of comprehensive cost-benefit analysis prior to investment, and curriculum misalignment underscore the importance of organizational readiness and ongoing support. For VR to be effectively integrated, it must not only align with educational goals but also be supported by infrastructure and a culture of collaboration, both within institutions and with external partners. Given the small, purposively selected sample, these findings are best understood as exploratory and hypothesis-generating. They suggest directions that warrant testing in larger and more diverse studies, illustrating the value of a holistic approach that bridges individual enthusiasm with structured institutional frameworks.

## Supplementary Information

Supplementary Material 1.

## Data Availability

The data supporting the findings of this study are available from the corresponding author upon reasonable request. While the data are not sensitive, they include information that could potentially identify participants. To protect privacy, the data are not publicly available but can be shared under conditions that ensure anonymity and compliance with ethical guidelines.

## Abbreviations

AR: Augmented Reality
DOI: Diffusion of Innovations
HMD: Head-Mounted Display
MR: Motivated Reasoning
VR: Virtual Reality
