# Supplementary material for: Organizational dynamics and individual motivations in VR integration in higher education
Source: BMC Med Educ. 2026 Jul 9;26:1567. doi: 10.1186/s12909-026-09911-3 (PMC13632227; doi:10.1186/s12909-026-09911-3)
Supplement: Supplementary file 1 [file 12909_2026_9911_MOESM1_ESM.docx]

**Introduction of VR**
I'm curious about how VR was introduced in your organization. Can you share the journey you had from considering VR to trying it out?

- How was VR initially brought to your attention?
- Can you describe the initial assessments made regarding VR for educational purposes?
- Who was involved in the decision-making process about using VR, and how was this decision made?
- What aspects were particularly emphasized during the decision-making process when VR was chosen?

**Choice of VR**
What made you decide to go with VR technology? Did you consider other alternatives, and what ultimately led to VR being selected?

- Was VR technology initially compared to other approaches or educational methods? If so, what tipped the scale in favour of VR?
- Were any preliminary studies or analyses conducted to understand the needs and expectations of end users, and if so, how were they carried out?
- What were your expectations for VR before you began the process, and how did you perceive others' expectations?

**Selection of VR Equipment and Content**
Can you explain how you decided on the type of VR equipment to use and the content to include?

- What was the reasoning behind choosing this specific VR equipment?
- Which content was deemed particularly relevant for your courses or study programs?
- Can you describe the process that led to selecting an external partner?
- What considerations were made regarding costs versus potential educational value (cost-benefit)?

**Collaboration in Developing VR Solutions**
What has your experience been with collaboration, both internally and externally, in developing VR solutions?

- How was the collaboration with VR providers or internal development teams organized, and what were your experiences with this collaboration?
- How was course content adapted or developed for the VR format, and how did you find the collaboration surrounding this?

**Implementation of VR**
I'm interested in hearing about how you implemented, or attempted to implement, VR in education.

- How was the implementation of VR planned and executed?
- How was VR integrated into existing learning platforms, curricula, or syllabi?

**Training and Support Services**
Can you describe how training in the use of VR technology was organized and what support services were provided for both technical and pedagogical support?

- How were end users trained in using the VR technology?
- What support services were established for technical and pedagogical assistance?

**Experiences with VR in Education**
What have your concrete experiences been with the use of VR? Do you have specific examples of how it has been utilized?

- Can you provide examples of how VR has been used in specific courses or projects?
- What observations have you made regarding how VR in teaching has been received by teachers, students, and others involved?
- What are your thoughts on using VR in your education programs, considering current needs and learning objectives, based on your experiences so far?

**Evaluation of VR**
How have you gone about measuring and assessing the impact of VR in education? What are the main findings?

- How do you evaluate the effectiveness of VR in educational contexts (methodology)?
- What were the key findings of these evaluations?

**Future of VR**
What are your thoughts on the future of VR in your organization? Do you have specific plans to develop or adjust its use?

- How do you plan to further develop or adjust the use of VR?
- Are there plans to expand the use of VR to other areas, and if so, which ones?
- What areas for improvement have you identified for the future use of VR?

**Long-term Vision for VR**
Can you share your long-term vision for the use of VR? How do you envision it influencing future learning experiences in your organization?

- How does VR fit into the long-term strategic plan for education?
- Do you foresee VR impacting future learning experiences, and if so, in what way?
- What advice would you give to other educational institutions considering the adoption of VR in education?

**Additional Thoughts or Perspectives**

Is there anything else you would like to add about the use of VR in education, something we haven’t covered yet, or any final reflections you have?

- Are there other aspects of using VR in education that haven’t been mentioned that you would like to highlight?
- Do you have any concluding reflections or insights based on your experience with VR in education?
